# Long COVID clinics and services offered by top US hospitals: an empirical analysis of clinical options as of May 2023

**DOI:** 10.1186/s12913-024-11071-3

**Published:** 2024-05-30

**Authors:** Alyson Haslam, Vinay Prasad

**Affiliations:** 1 University of California San Francisco, 550 16th St, 2nd Fl, San Francisco, CA 94158 USA; 2 Department of Epidemiology and Biostatistics, UCSF Mission Bay Campus, Mission Hall: Global Health & Clinical Sciences Building | 550 16th St, 2nd Fl, 94158 San Francisco, CA USA

## Abstract

**Background:**

The economic and health burden of COVID-19 has transformed the healthcare system in the US. Hospitals have adapted to the heterogeneity in long COVID symptoms, and the sheer number of people affected by this condition, by building long COVID centers and programs.

**Objective:**

We sought to describe characteristics, services, and clinical trials of long COVID centers at top US hospitals.

**Design:**

Cross-sectional analysis.

**Participants:**

Long COVID treatment programs or centers at top US hospitals.

**Exposures:**

Frequency of long COVID centers, eligibility for being treated, the services they provide, specialist to whom the patients may be referred, and the long COVID clinical trials in which these hospitals participate.

**Findings:**

Most top hospitals in the US (*n* = 43/50; 86%) offer long COVID services. 65% (28/43) did not describe the services provided. 12 (28%) required a referral from a primary care physician. The most common services were meeting with a team member (*n* = 20; 47%), ordering lab and/or radiology services (*n* = 8; 18.6%), and administering a physical exam (*n* = 7; 16%). 7 (16%) centers/programs treated only adults; 5 (12%) treated both adults and children, and 31 (72%) did not specify. The most common specialists described were psychology (*n* = 25; 58%), neurology (*n* = 25; 58%), and pulmonary (*n* = 24; 56%). Sixty-three trials (of 134 long COVID clinical trials) had at least one top hospital listed as a study site. The median number of clinical trials that each hospital sponsored or was a study site was 2 (interquartile range: 1, 3).

**Conclusions and relevance:**

We find that services offered at long COVID clinics at top hospitals in the US often include meeting with a team member and referrals to a wide range of specialists. The diversity in long COVID services offered parallels the diversity in long COVID symptoms, suggesting a need for better consensus in developing and delivering treatment.

## Background

Long COVID is a concerning condition with a broad array of symptoms, varying and debated symptom duration, and currently ill-defined prognosis, which has led to multiple definitions being implemented [1, 2]. The number of symptoms has been estimated to be as high as 200, (https://pubmed.ncbi.nlm.nih.gov/36639608/ ) and because of the complexity, researchers and healthcare providers have sought to refine the list of symptoms through a more data-driven approach. This list includes symptoms relating to smell/taste, malaise, cough, brain fog, thirst, palpitations, chest pain, fatigue, sexual desire/capacity, dizziness, and/or gastrointestinal disturbances. (https://www.ncbi.nlm.nih.gov/pmc/articles/PMC10214179/ )

The potential health and economic impacts of COVID-19 are great. For example, the CDC has reported that nearly one in five people in the US with a prior history of COVID-19 continue to suffer from long COVID symptoms [3]. Moreover, the economic costs, due to reduced quality of life, lost earnings, and increased medical spending has been estimated to be $3.7 trillion dollars, or 17% of the US gross domestic product for 2019 [4]. According to some analysts, long COVID is poised to transform both the health of the population and the healthcare industry [5].

The heterogeneity in long COVID symptoms makes finding appropriate help and developing appropriate treatment plan(s) difficult. The Administration for Community Living, a division of the US Department of Health and Human Services, has outlined navigational steps for people who are seeking help and resources for disabilities due to long COVID [6]. To what extent treatments will be tailored for individuals, or formed on the basis of prospective, randomized population data remains unknown.

Given the growing demand for long covid services, hospitals have responded with a mixed effort of research and treatment. Often services begin in the primary care setting, with an eventual referral to a long-COVID clinic. ﻿(https://www.ncbi.nlm.nih.gov/pmc/articles/PMC10165471/ )﻿ The services provided can range from psychological to physical rehabilitation, to treatment of comorbidities. Many hospitals have established and advertised long COVID programs and clinics, yet little is known about the frequency and characteristics of these offered services.

We sought to characterize publicly available information on the frequency and nature of long COVID services offered at top hospitals in the US.

## Methods

The purpose of the present investigation was to characterize the frequency with which Newsweek’s top 50 hospitals advertise, as of May 17, 2023, the presence of long COVID clinics, centers or programs, and the characteristics of those offerings [7].

For each of the hospitals, we did a Google search of long COVID centers, clinics, or programs, by searching the hospital name and “long COVID center”. We viewed the first 10 search results for program/clinic websites/webpages, and if we found no website/webpage, we searched for news articles discussing a program or clinic.

For each long COVID program, we abstracted data pertaining to the eligibility requirements (proof of testing positive, age, referral requirement, duration of symptoms) of the program or clinic, evaluation, testing, and examination components, follow-up activities, and areas of specialty. For areas of specialty, we noted specialties on the list, if one was provided, or searched the specialties of physicians affiliated with the center/program. We also abstracted data on the department under which the center or program is housed. If the department was not specifically stated and there was a sitemap breadcrumb trail, we assumed that the center or program was housed under the department in the breadcrumb trail. Otherwise, we listed it as not indicated.

We then searched clinicaltrials.gov (initial search on July 3, 2023 and updated on January 22, 2024) for long COVID clinical trials, using the search condition of long COVID and restricted to trials in the US. We searched the list of trials to see if and how many trials each hospital was an affiliated sponsor and/or study location. We matched on either the name of the hospital or the name of the university affiliated with the hospital.

We calculated frequencies and percentages of center/program characteristics and number of clinical trials each hospital was listed as a participant. We used package ‘usmap’ to map distributions of long COVID centers and long COVID clinical trials, by state. All analyses were conducted in R statistical software, version 4.2.1.

In accordance with 45 CFR § 46.102(f), this study was not submitted for University of California, San Francisco institutional review board approval because it involved publicly available data and did not involve individual patient data.

## Results

**Fig. 1:**
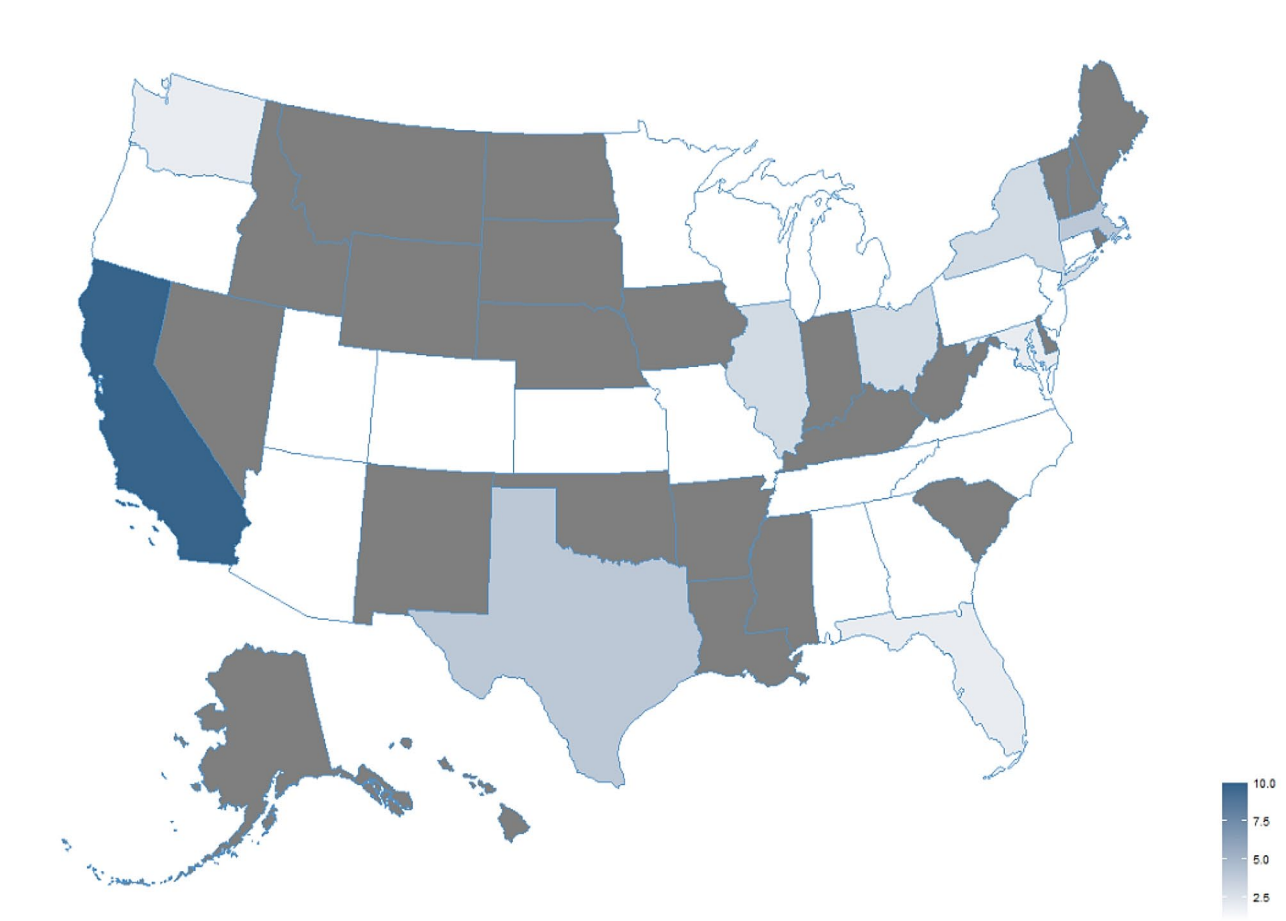
Distribution of long COVID centers/programs among the top 50 US hospitals [7]. Gray colored states did not have a hospital on the top 50 list

We found that, among the top US 50 hospitals per Newsweek’s, most (*n* = 43; 86%) offer long COVID services. Figure 1 shows the distribution, by state, of long COVID centers/programs among the top 50 US hospitals. There were 26 different states represented in the top 50 hospitals, and each one had at least one long COVID center/program present (median of 1). California had the most long COVID centers at the top hospitals (*n* = 10). Most hospitals with long COVID services referred to themselves as clinics (*n* = 29; 67%), while 13 (30%) referred to themselves as a program and 1 (2%) was too vague to determine. Thirty-three (67%) centers had an official website describing their services, and 10 were described in an article or blog.

Table 1 shows the characteristics of long COVID programs and services at the top hospitals with a long COVID clinic or program. Of the 43 hospitals with long COVID services, the services described were a meeting with one of the long COVID treatment team members (*n* = 20; 47%), ordering lab and/or radiology services (*n* = 8; 18.6%), administering a physical exam (*n* = 7; 16%), assessing functional status (*n* = 6; 14%), prescribing medication (*n* = 3; 7%) and/or; referral to counseling (*n* = 4; 9%) or rehabilitation services (*n* = 3; 7%). Fifteen (35%) did not describe their specific services.

**Table 1:** Long COVID centers among the top US 50 hospitals, according to Newsweek (*N* = 43) [7]^*^

Clinic or program (%)	
Clinic	30 (60.0)
Program	13 (26.0)
Serviced provided (could be more than one; %)	
Meet the team	20 (46.5)
Lab radiology	8 (18.6)
Physical exam	7 (16.3)
Screening	6 (14.0)
Management group	5 (11.6)
Counseling	4 (9.3)
Medication	3 (7.0)
Rehabilitation	3 (7.0)
No information on service provided (%)	15 (34.9)
Eligibility criteria	
Proof of positive COVID test (%)	
Yes	19 (44.2)
No	4 (9.3)
Not indicated	20 (46.5)
Population (%)	
Adult	7 (16.3)
Adult and children	5 (11.6)
Not indicated	31 (72.1)
Referral (%)	
Yes	12 (27.9)
No	5 (11.6)
Not indicated	26 (60.5)
Minimum duration of symptoms (%)	
4 weeks	11 (25.6)
12 weeks	6 (14.0)
6 weeks	2 (4.7)
8 weeks	1 (2.3)
Not indicated	23 (53.5)
No eligibility information provided (population, referral requirement, duration of symptoms; %)	10 (23.3)
Specialist services (could be more than one; %)	
Neurology	25 (58.1)
Psychology	25 (58.1)
Pulmonary	24 (55.8)
Rehabilitation specialist	23 (53.5)
Cardiology	22 (51.2)
Infectious disease	14 (32.6)
Rheumatology	14 (32.6)
Nephrology	9 (20.9)
Endocrinology	7 (16.3)
Gastrointestinal	7 (16.3)
Sleep medicine	7 (16.3)
Otolaryngology	6 (14.0)
Dermatology	5 (11.6)
Other specialist	24 (55.8)
No information on specialist services (%)	10 (23.3)
Source of center/program information (%)	
Center/program website	33 (66.0)
Hospital blog	4 (8.0)
Hospital article	3 (6.0)
Article	2 (4.0)
University article	1 (2.0)
Department housing clinic or program (%)	
Not indicated	28 (65.1)
Multiple	5 (11.6)
Pulmonary	4 (9.3)
Infectious disease	3 (7.0)
Rehabilitation	2 (4.7)
Neurology	1 (2.3)
Number of long COVID clinical trials per hospital (Median (IQR))	2 (1,3)

*7 hospitals did not have internet presence of a long COVID center or program

We found 19 (44%) required documentation of a prior positive COVID-19 test; 4 (9%) did not; and 20 (47%) did not specify. We found 7 (16%) treated only adults; 5 (12%) treated both adults and children, and 31 (72%) did not specify. There were 12 (28%) centers that required a referral from a primary care physician; 5 (12%) did not; and 26 (61%) did not specify. There were 11 (26%) centers that specified that long COVID symptoms needed to have occurred for at least four weeks; 6 (14%) specified 12 weeks; two (5%) specified six weeks; one (2%) specified eight weeks, and 23 (54%) did not specify. Ten (23%) hospitals did not describe any eligibility criteria.

The most common specialists described were psychology (*n* = 25; 58%); neurology (*n* = 25; 58%); pulmonary (*n* = 24; 56%); cardiology (*n* = 22; 51%); rheumatology (*n* = 14; 33%); and infectious disease (*n* = 14; 33%). The department that the clinic or program resides in is most often not indicated (*n* = 28; 65.1%), and if it is, it is often a joint collaboration between more than one department, often including pulmonary, infectious disease, and/or rehabilitation (*n* = 5; 11.6%).

**Fig. 2:**
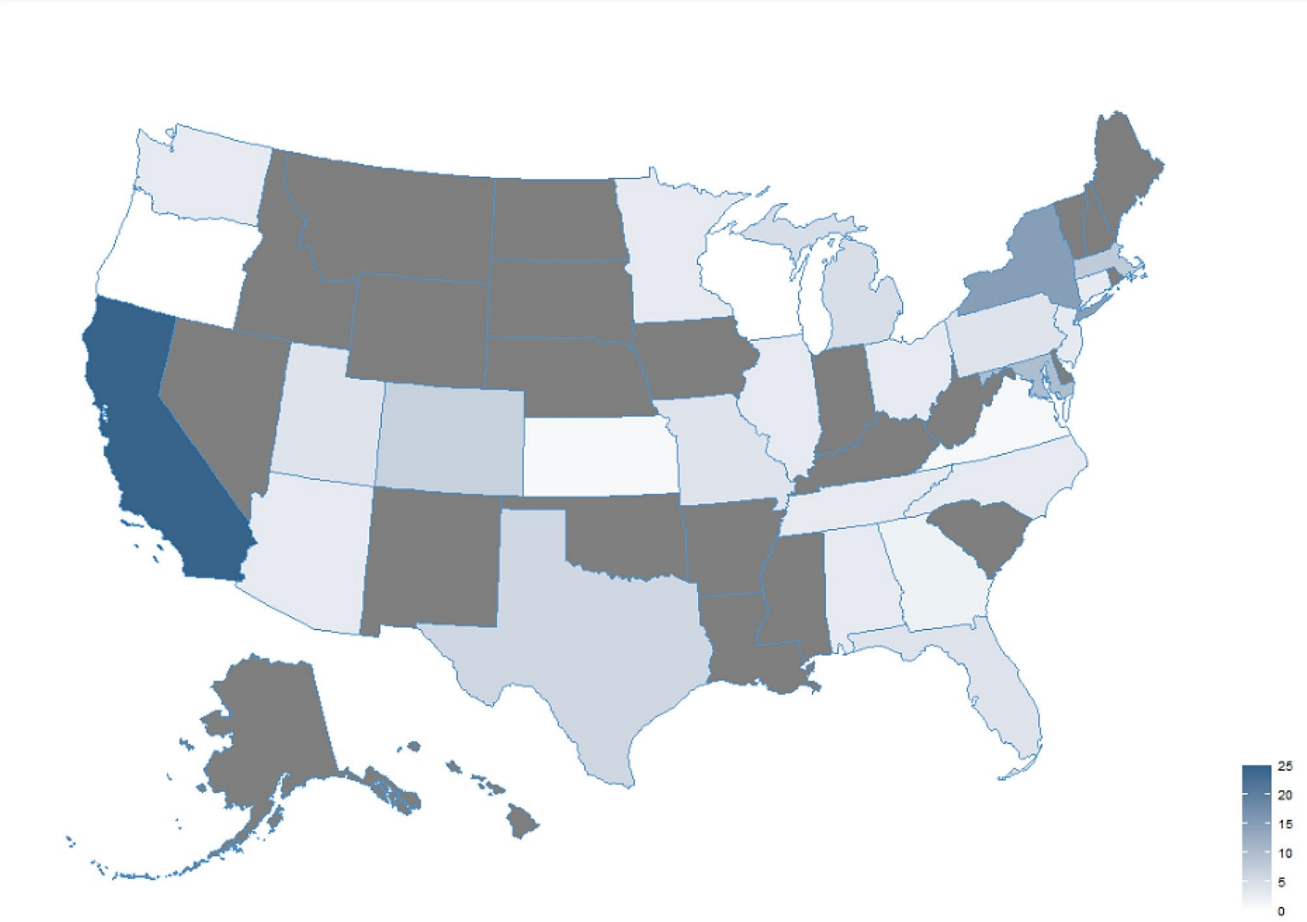
Distribution of long COVID clinical trials among long COVID centers/programs in the top 50 US hospitals [7]. Gray states did not have a hospital on the top 50 list

There were 134 clinical trials in the US for long COVID. Sixty-three trials had at least one of the top 50 hospitals listed as a study sponsor and/or study location. The median number of clinical trials that each hospital sponsored or was a study site was 2 (interquartile range: 1, 3). The median number of long COVID clinical trials for top hospitals, by state, was 3 (interquartile range: 1, 5). Among long-covid trials, 35/134 (26.1%; 18/63 [28.6%] of the hospitals with long COVID centers) were observational, 72/134 (53.7%; 36/63 [57.1%] of the hospitals with long COVID centers) were randomized. Figure 2 shows the distribution, by state, of long COVID clinical trials among long COVID centers/programs in the top 50 US hospitals.

## Discussion

Top US hospitals, as recognized by a popular magazine ranking, already frequently provide long COVID services and advertise them with varying levels of detail. The services they provide often include meeting with a team member at the clinic and referrals to a wide range of specialists. They also include clinical trial opportunities but appear not to be limited to trials. These findings, to our knowledge, are the first in characterizing the services offered at long COVID treatment centers at top hospitals. Others have characterized long COVID centers based on a convenience sample of surveys sent to long COVID centers earlier in the COVID-19 pandemic. (https://onlinelibrary.wiley.com/doi/https://doi.org/10.1002/pmrj.12766 ) Moreover, healthcare models have been developed with the intent to adaptively address needs of the healthcare organization and the patient population of the respective organization. (https://www.ncbi.nlm.nih.gov/pmc/articles/PMC10165471/ ; https://www.ncbi.nlm.nih.gov/pmc/articles/PMC8594397/ ; https://pubmed.ncbi.nlm.nih.gov/36324552/ )

Here we present several key findings from our research. One overall finding is that 86% of hospitals offer long COVID services, which is the majority of hospitals in our dataset. Neurology, psychology, and pulmonary were the three most commonly mentioned specialists to where long COVID centers might provide a patient referral. We have previously reported that the most common symptoms ascribed in long COVID study definitions were fatigue, shortness of breath, cognitive impairment, and joint/muscular pain [1], which are commonly seen by these specialists.

There has been a great deal of research into the biological and pathological underpinnings of long COVID. The results of these studies are inconsistent in whether there are differences in pathological measurements between people who have had COVID-19 and those who have not. (https://www.ajnr.org/content/ajnr/44/5/517.full.pdf ) https://journals.physiology.org/doi/pdf/https://doi.org/10.1152/ajpheart.00335.2022 ) The results of these comparison studies highlight the difficulty in developing treatment options for a condition that is not only heterogeneous in symptoms, but also does not always manifest with pathologic differences from those without COVID-19. Ideally, services offered at the clinics would be based on known scientific understanding.

7% report that they will offer some medications as therapy, yet, to our knowledge, no treatment has received US FDA approval for long COVID. Medications may be prescribed to treat related conditions, and adequately powered randomized trials should be conducted to assess whether treatment options are effective in patients with long COVID.

We found 134 long COVID studies registered on clinicaltrials.gov. Of these studies, 54% were randomized, but just over one-quarter were observational. These percentages were similar for studies sponsored/conducted by top hospitals with long COVID centers vs. not sponsored/conducted. Observational studies are helpful for studying the long-term prognosis, but in order to find effective preventive and treatment strategies, randomized trials are essential. In other words, we need randomized trials, not “random” care, to determine appropriate care in the shortest amount of time [9].

Others have informally assessed the geographical distribution of long COVID treatment centers, nationally. From their assessment, there were two notable observations [10]. First, most long COVID care is offered by physical therapy and rehabilitation centers. Two, there appears to be a mismatch between long COVID clinic locations and the prevalence of long COVID in an area. While our results are limited to long COVID centers at top US hospitals, our findings indicate that most services encompass meeting with team members at the clinic and referrals to specialists. Rehabilitation services are specifically mentioned as initial services on only a few clinic/program websites but are common outcomes of referrals. However, our findings indicate that there are large geographical regions with limited care to long COVID centers or programs at top hospitals.

The finding that most (65%) clinics and programs do not indicate the department that the program or clinic resides suggests that many clinics are independent departments and that hospitals are not only heavily invested in providing immediate care, but also care well into the future. Indeed, hospitals have had to pivot and adapt to changing markets during and since the COVID-19 pandemic began [11].

A concern with the high number of specialist referrals at these clinics is the already long wait time for patients to see a specialist, where non-COVID patients can wait up to a median of about 2.5 months to see a specialist [12, 13], but a backlog due to delayed care during the pandemic could likely increase these times [11]. Anecdotal data suggest that patients who attend long COVID clinics also experience a long wait time before they can be seen and sometimes make long drives for their appointments [14]. The time to see a healthcare provider, and especially a specialist, could further increase given the number of people being diagnosed with this condition and referred to follow-up care. Non-traditional modes of healthcare delivery, including telemedicine, may be options for increasing access to healthcare for patients with long COVID, including pregnant, pediatric, older individuals as well as those from racial/ethnic minority groups. (https://pubmed.ncbi.nlm.nih.gov/37419538/ )

Our analysis has several limitations. For one thing, we were using websites that may not have reported the most current information. Related, the websites may not have provided all information related to the care they provided. Our results are from the view of the patient who may be seeking for long COVID care with the help of general Google searches. Another limitation is that our results are not generalizable to long COVID centers at-large. We used a list of the top hospitals, which would theoretically result in a sampling of top long COVID centers, with the ability to deliver expert care across a broad spectrum of medical specialties. Additionally, a different list of top hospitals, based on other metrics, could result in different characteristics of long COVID centers.

## Conclusion

We find that services offered at long COVID clinics at top hospitals in the US often include meeting with a team member and referrals to a wide range of specialists. The specialists most often include psychological, neurological, or pulmonary. The diversity in long COVID services offered parallels the diversity in long COVID symptoms, suggesting a need for better consensus in developing and delivering treatment.

## Data Availability

The datasets used and/or analysed during the current study available from the corresponding author on reasonable request.
